# Comparative Analysis of Letrozole and Estradiol Valerate PCOS Models: Reproductive and Metabolic Outcomes with and Without High-Fat Diet

**DOI:** 10.3390/biology14060592

**Published:** 2025-05-23

**Authors:** Xóchitl Acuña Escalona, Rocio Sarahy Ayala, Karla Cortez, Sophie Fernández Sánchez, Teresa Tomé-Dehesa, Verónica Díaz-Hernández, Carlos Larqué, Rene Escalona

**Affiliations:** 1 Laboratory of Embryology and Genetics, Departamento de Embriología y Genética, Facultad de Medicina, Universidad Nacional Autónoma de México, Ciudad de México 04510, Mexico; andmara24@gmail.com (X.A.E.); sarahy.ayala.martinez@gmail.com (R.S.A.); karlacortezh99@gmail.com (K.C.); mc25fess4708@facmed.unam.mx (S.F.S.); tdmariateresa@gmail.com (T.T.-D.); diazveronica@comunidad.unam.mx (V.D.-H.); calvelazquez@facmed.unam.mx (C.L.); 2 Posgrado en Cinecias Biológicas, Universidad Nacional Autónoma de México, Ciudad de México 04510, Mexico

**Keywords:** polycystic ovary syndrome, letrozole, estradiol valerate, high-fat diet, insulin resistance

## Abstract

Polycystic ovary syndrome (PCOS) is a prevalent endocrine disorder in reproductive-aged women, characterized by hyperandrogenism, oligoanovulation, and polycystic ovarian morphology. Despite its classification as a reproductive disorder, PCOS is closely associated with metabolic dysregulation, including insulin resistance and obesity. An ideal animal model for PCOS should replicate both reproductive and metabolic features of the condition. In this study, we compared two widely used postnatal PCOS models (letrozole and estradiol valerate [EV]) administered alone or in combination with a high-fat diet (HFD), assessing their ability to induce both the reproductive and metabolic features. Letrozole treatment led to significant weight gain and increased visceral adiposity, effects that were amplified by HFD. Conversely, EV treatment showed a tendency toward reduced body mass. While neither model significantly altered fasting glucose levels, letrozole combined with HFD impaired glucose tolerance, supporting its role in metabolic dysfunction. Hyperandrogenism was more consistently induced by letrozole compared to EV, aligning with clinical PCOS phenotypes. Both treatments disrupted estrous cyclicity and induced polycystic ovarian morphology, though metabolic disturbances were more pronounced in the letrozole model. These findings suggest that letrozole, particularly in combination with HFD, provides a more consistent model for studying both the reproductive and metabolic facets of PCOS.

## Simple Summary

Polycystic ovary syndrome (PCOS) is a common condition in women that affects hormones and periods, and can make it harder to get pregnant. It is also linked to problems like weight gain and difficulty processing sugar. Scientists use animal models to study PCOS, but not all models show both the hormone and metabolism problems seen in people with PCOS. In this study, two common ways to create PCOS in animals using hormone treatments—letrozole and estradiol valerate (EV)—with or without a high-fat diet were tested. It was found that letrozole, especially when combined with a high-fat diet, caused weight gain, hormone imbalances, and changes in the ovaries similar to PCOS in humans. On the other hand, EV had fewer of these effects. This suggests that the letrozole plus high-fat diet model is the most useful for studying both the hormone and metabolism issues of PCOS.

## 1. Introduction

Polycystic Ovary Syndrome (PCOS) is a condition characterized by an excess production of male sex hormones. PCOS is one of the most common endocrine disorders in women; it is estimated to affect 5–20% of women of reproductive age worldwide [1]. This disorder is characterized by a triad of (a) hyperandrogenism, (b) oligomenorrhea, and (c) polycystic ovarian morphology, with hyperandrogenism being the most important sign and directly associated with the metabolic complications often observed in women with PCOS [2]. For example, women with PCOS have a higher incidence of insulin resistance (IR) [1,2]. Additionally, patients with PCOS have an increased risk of developing glucose intolerance and type 2 diabetes mellitus (T2DM). However, one of the main comorbidities associated with PCOS is infertility, affecting up to 70% of women with PCOS, compared to 16% in women without a PCOS diagnosis [3]. Furthermore, PCOS and its associated metabolic disorders are linked to a higher likelihood of pregnancy complications, such as increased rates of miscarriage [4]. Overall, PCOS is the most common cause of female infertility worldwide [1].

Due to the complexity and heterogeneity of PCOS, it is difficult to establish a clear mechanism underlying the development of the syndrome; genetic, endocrine, and even environmental factors have been identified [2]. However, as previously mentioned, the central element in the development of the metabolic and reproductive disorders in PCOS is hyperandrogenism. Women with PCOS exhibit hyperandrogenism primarily due to the overproduction of steroid hormones in theca cells, resulting from an increase in the production of enzymes in the biosynthetic pathway of these hormones (CYP17A1, CYP1A1, CYP11A, and HSD17B6) [5,6]. Additionally, patients with PCOS present alterations in the secretion of GnRH in the hypothalamus, leading to an increased LH/FSH ratio, which stimulates androgen secretion and causes follicular arrest [6]. Finally, women with PCOS have a higher secretion of insulin (compared to healthy women of the same BMI) and lower hepatic insulin clearance, resulting in hyperinsulinemia [7,8]. As a consequence of hyperinsulinemia, there is an increased secretion of androgens by theca cells, as well as reduced hepatic synthesis of sex hormone-binding globulin (SHBG). Together, these elements promote the development of a hyperandrogenic environment.

Given the importance of PCOS, various animal models have been developed to study it. These models can be created through various manipulations and/or pharmacological protocols and may exhibit two or more of the symptoms observed in PCOS [9]. These treatments can be administered orally, subcutaneously, via implantation, at different stages of development, and for varying administration periods [10]. Thus, there are multiple models and administration regimens to simulate PCOS, resulting in a wide heterogeneity of phenotypes. One of the most common models is the administration of letrozole (LET), a highly specific and selective competitive inhibitor of the enzyme aromatase. Treatment with LET blocks the conversion of testosterone into estradiol, thereby decreasing the estradiol/testosterone ratio. LET can be administered either through oral gavage or via subdermal implants. Furthermore, this model has been used in addition to a high-fat diet to enhance the metabolic disorders often observed in patients with PCOS [11]. On the other hand, estradiol valerate (EV, a long-acting form of estrogen) is also commonly used to induce PCOS in animal models. This model is typically established by administering a single dose of 2–4 mg of EV subcutaneously to prepubescent rats [12]. One study observed that after 20 days of EV injection, all the rats remained in continuous estrus, and their ovaries displayed large cyst-like follicles with severe atresia. Additionally, LH levels decreased following the EV injection. Administering 2 mg of EV resulted in increased levels of testosterone and estradiol. The rats’ body weight remained similar to that of the control group, but there was a noticeable reduction in FSH, progesterone levels, and corpora lutea [13].

Furthermore, additional chemical models of PCOS include the administration of androgens (testosterone, dihydrotestosterone, dehydroepiandrosterone, etc.), progesterone receptor antagonists, high-fat diet (HFD) feeding, bisphenol A exposure, among others [9]. However, to date there are no models that fully replicate the entire spectrum of PCOS characteristics. They often lack consistency in metabolic dysfunction (e.g., insulin resistance; obesity) or do not mimic the full hormonal and ovarian profile seen in patients. A more refined rat model is needed, one that better integrates environmental factors and developmental timing to reflect the syndrome’s complexity. Such a model would enhance translational relevance, allowing for improved mechanistic studies and the development of targeted therapies with higher predictive value for human clinical outcomes. Thus, in this paper we aim to compare two PCOS models, LET and EV, with and without the administration of an HFD. The metabolic and reproductive phenotypes of these models were evaluated and compared using Wistar rats.

## 2. Materials and Methods

### 2.1. PCOS Induction Models

To establish a letrozole model, 12-week-old Wistar female rats were administered letrozole (Santa Cruz Biotechnology, CAS 112809-51-5) at a dose of 1 mg/kg bodyweight, dissolved in 1% carboxymethylcellulose (CMC, Santa Cruz Biotechnology, Dallas, TX, USA, CAS 9004-32-4), for 21 consecutive days through oral gavage. This postnatal regime of letrozole has been used previously to induce PCOS symptoms [14,15]. For the EV model, 21-day-old Wistar female rats were subcutaneously injected with 4 mg of estradiol valerate (Sigma Aldrich, St. Louis, MO, USA, CAS 979-32-8) dissolved in sesame oil (SO, Santa Cruz Biotechnology CAS 8008-74-0) and were evaluated 84 days later. This prepuberal model has also been previously been reported to induce PCOS [16,17]. Rats were randomly divided into three experimental groups for each of the two models tested. For the letrozole model: CMC, LET, and LET + HFD; for the estradiol valerate model: SO, EV, and EV + HFD. Rats were housed in acrylic cages (45 × 30 × 20 cm) in pairs under controlled temperature (22 ± 2 °C). For 12 weeks (immediately after weaning at 3 weeks of age), the animals had ad libitum access to water and food, according to their experimental group. Body weight gain and caloric intake were monitored weekly in all groups. The committee for ethical evaluation at the Facultad de Medicina, UNAM approved experiments according to international guidelines for the ethical use of animals (protocol code 015-2023). All procedures were designed to minimize the number of animals used and their suffering.

### 2.2. Diets

Animals allocated to the CMC, LET, SO, and EV groups were fed standard rodent chow (Laboratory Rodent Diet 5001, LabDiet^®^, Arden Hills, MN, USA). The control (CTR) diet had a caloric output of 3.36 kcal/gram, with 13%, 28%, and 59% calories provided by fats, protein, and carbohydrates, respectively. Animals allocated to the LET + HFD and EV + HFD groups were given a diet manufactured in the Department of Embryology and Genetics of Facultad de Medicina, UNAM. In brief, for every 100 g of HFD, 49.5 g of ground Laboratory Rodent Diet 5001 pellets, 35.0 g of lard and 15 g of lyophilized egg albumen were thoroughly mixed and refrigerated before being administered to the animals [18]. The HFD had a caloric output of 5.41 kcal/gram, with 62%, 20%, and 18% calories provided by fats, protein, and carbohydrates, respectively.

### 2.3. Intraperitoneal Glucose Tolerance Test (IPGTT) and Insulin Tolerance Test (ITT)

One week before the end of the experimental period, the animals were fasted for 12 h to determine fasting plasma glucose (FPG) and to perform the IPGTT and ITT. For IPGTT, 2 g glucose/kg bodyweight were administered intraperitoneally; for the ITT, 0.2 IU/kg of human recombinant insulin (Humulin, Lilly, Indianapolis, IN, USA) was administered. Blood glucose levels were measured at 15, 30, 60, 90, and 120 min after the intervention using a handheld blood glucose monitor (Accu-Chek Instant, Roche, Basel, Switzerland).

### 2.4. Estrus Cycle Analysis

Estrous cycle determination was carried out by vaginal smears, which were obtained for 14 days starting on postnatal day 60. Vaginal smears were collected by gently inserting a wire loop into the rat’s vagina. The material was smeared onto microscope slides and stained with hematoxylin–eosin. The estrous cycle phase was determined according to the predominant cell types in each slide observed under a microscope [19]. Briefly, proestrus is characterized by an abundance of nucleated cells and few cornified cells (5:1), estrus is characterized by the presence of both cornified and nucleated cells (1:1), metestrus is characterized by the abundance of leukocytes and few nucleated cells (10:1), and diestrus is characterized by abundant leukocytes and few nucleated cells (3:1); representative images of vaginal cytology are provided on Supplementary Figure S2. Rats were considered regular if they exhibited 4–5-day cycles with the correct progression between phases. Thus, rats with longer phases or those that presented an incorrect progression of stages were considered to have irregular cycles.

### 2.5. Tissue Extraction

At the end of each experiment, all rats were euthanized after a 12 h fast with a pentobarbital overdose (Pisabental, Aranda; 100 mg/kg). Blood was extracted via cardiac puncture and was collected in EDTA-containing tubes. Plasma was separated by centrifugation and stored at −80 °C until analyzed. Gonadal tissue was removed and washed in ice-cold phosphate-buffer saline (PBS), and then fixed in 4% paraformaldehyde in PBS at 4 °C. After fixation, the tissues were dehydrated and embedded in paraffin for sectioning.

### 2.6. Histological Analysis

To study histological alterations in the ovaries, an analysis of hematoxylin–eosin-stained paraffin sections was performed in all groups. Ovarian morphology was assessed in paraffin sections using one ovary selected at random from each animal. The ovaries were cut into 10 μm thick sections; every tenth section was collected on glass slides. A total of 4–5 sections per ovary were analyzed.

### 2.7. Hormone Determination

Plasma estradiol (ER1507), testosterone (EU0400-HS), and insulin (ER1113) were determined using an ELISA (FineTest^®^, Wuhan, China), according to the manufacturer’s instructions.

### 2.8. Statistical Analysis

**Figure 1:**
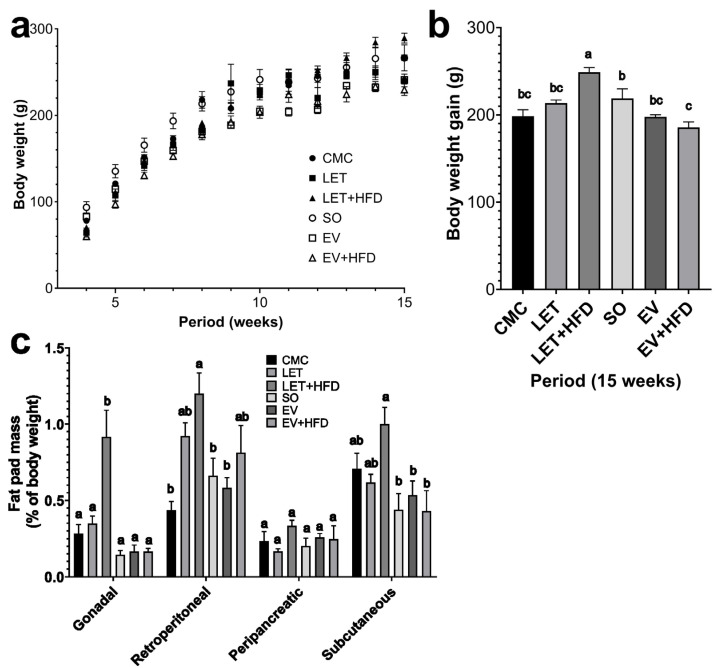
**Somatometric characterization of treated females during and after experimental treatment.** (**a**) Weekly body weight gain of females during the 15-week experimental period; (**b**) total weight gain at the end of the experimental period; (**c**) weight of adipose tissue pads normalized to body weight. Each point/bar represents mean ± SEM. Bars labeled with different letter scripts indicate statistically significant differences. Individual *p* values for each point in panel 1a are provided in supplemental material. CMC (*n* = 6), LET (*n* = 7), LET + HFD (*n* = 12), SO (*n* = 6), EV (*n* = 8) and EV + HFD (*n* = 7).

**Figure 2:**
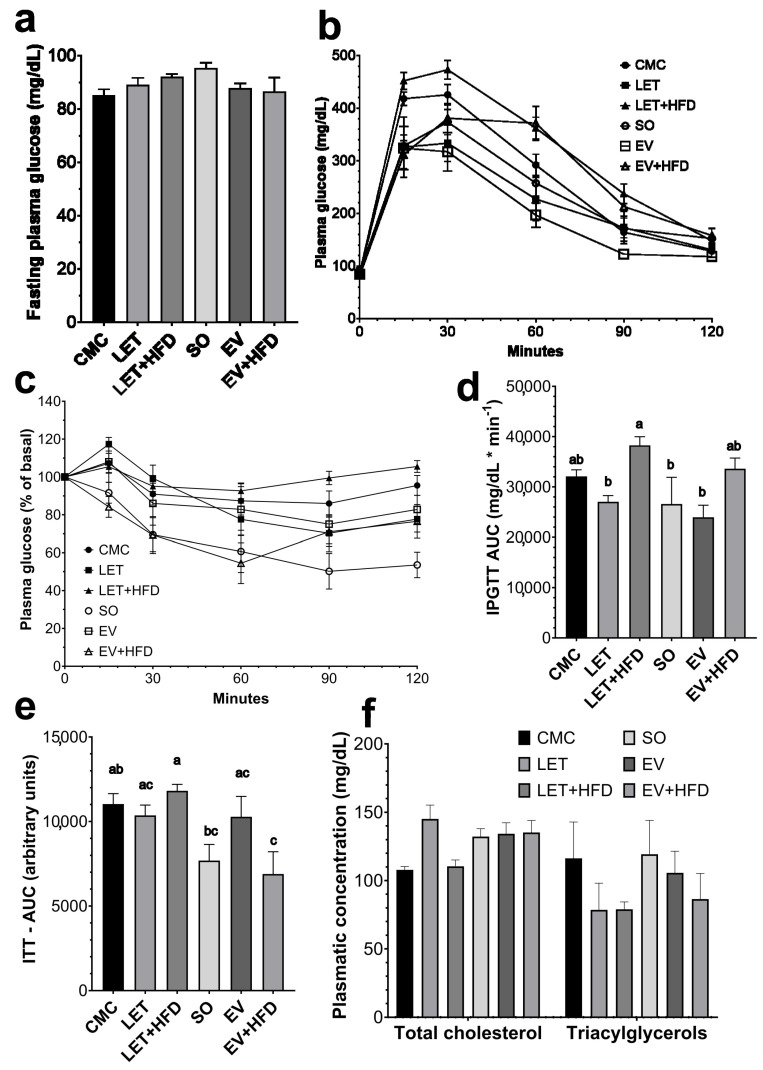
**Metabolic characterization of treated females after experimental treatment.** (**a**) Fasting blood glucose levels; (**b**) intraperitoneal glucose tolerance test (IPGTT); (**c**) insulin tolerance test (ITT); (**d**) area under the curve for the IPGTT; (**e**) area under the curve for the ITT; (**f**) plasma lipid profile. Each point/bar represents mean ± SEM. Bars labeled with different letter scripts indicate statistically significant differences. Individual *p* values for each point in panels (**b**,**c**) are provided in supplemental material. CMC (*n* = 6), LET (*n* = 7), LET + HFD (*n* = 12), SO (*n* = 6), EV (*n* = 8), and EV + HFD (*n* = 7).

GraphPad Prism 10.0 software was used for statistical analysis; data are presented as means ± standard error of the mean (SEM). A one-way ANOVA was used for multiple comparisons, followed by a post hoc Tukey test. Sample sizes are indicated in the figure legends, with *p* < 0.05 considered statistically significant. Individual *p* values for each point in Figure 1a and Figure 2b,c are provided in the Supplemental Materials.

## 3. Results

To compare the effects of two different PCOS models on metabolic and reproductive health parameters, we followed female Wistar rats for 15 weeks. Rats in the LET and LET + HFD groups displayed increased weight gain after letrozole administration, compared to the CMC group. This weight difference reached statistical significance by the end of the experimental period (16% and 20% for the LET and LET + HFD groups, respectively) (Figure 1a,b). Conversely, rats allocated to the EV and EV + HFD groups consistently exhibited reduced body weight compared to the SO group in the weeks following estradiol valerate administration. By the end of the experimental period, rats in the EV and EV + HFD groups were 10% and 14% lighter than their SO counterparts (Figure 1a,b). Additionally, when the total body weight gain across the experimental period was assessed, it was observed that, overall, LET + HFD gained the most body mass compared to the other treatments (Figure 1b). Conversely, the EV groups gained the least amount of body mass, regardless of the diet (Figure 1b). Thus, LET treatment induces increased body mass, and likely the metabolic alterations often associated with it. Furthermore, we attempted to determine whether both models altered the body composition in the experimental animals. As depicted in Figure 1c, LET + HFD group displayed an increase in the proportional weight of gonadal and retroperitoneal adipose tissue depots, compared to CMC and LET groups. However, there were no statistically significant differences between both in any of the remaining adipose depots. On the other hand, rats in the EV and EV + HFD groups exhibited similar adiposity compared to the SO group. Therefore, only LET + HFD treatment is capable of inducing increased body mass, due to an expansion of adipose tissue, while estradiol valerate did not modify the body composition of the experimental animals, even in the presence of an HFD.

To further determine whether either of the two models induced alterations in glucose homeostasis, we first measured fasting glucose levels. Regarding animals on the letrozole model, the LET + HFD group showed a tendency towards increased fasting plasma glucose levels, compared to the CMC group, without reaching statistical significance (Figure 2a). On the other hand, the EV and EV + HFD groups showed a tendency for decreased fasting glucose levels, though neither reached statistical significance (Figure 2a). Subsequently, an intraperitoneal glucose tolerance test (IPGTT) was performed on animals from both models. When compared to CMC, the LET group displayed a tendency towards a smaller curve. Conversely, the LET + HFD group displayed the opposite tendency, being significantly bigger than the LET group (Figure 2b,d). Similarly, neither the EV nor the EV + HFD groups displayed any significant change in glucose uptake (Figure 2b,d). However, LET + HFD had a significantly increased curve compared to EV. Insulin sensitivity was then assessed with an intraperitoneal insulin tolerance test. While no significant differences were observed within models (Figure 2c,e), the LET + HFD group had a reduced insulin sensitivity compared to EV + HFD (Figure 2c,e). Finally, plasma concentrations of total cholesterol and triacylglycerols were assessed to identify a possible alteration in lipid homeostasis due to diet or pharmacological treatment. Surprisingly, none of the treated groups displayed an increase in plasma lipid concentration (Figure 2f).

**Figure 3:**
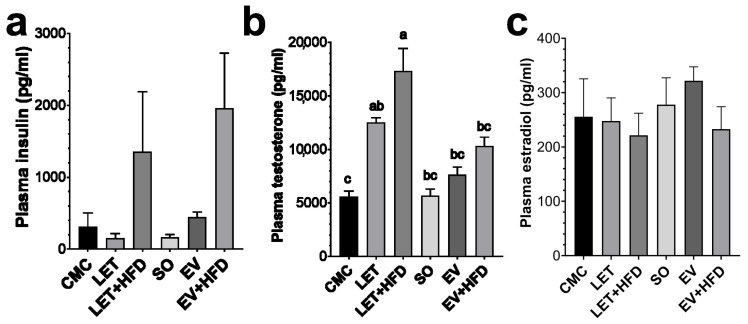
**Hormonal profile of experimental females**. (**a**) Fasting plasma insulin; (**b**) plasma testosterone concentrations; (**c**) plasma estradiol concentrations. Each bar represents mean ± SEM. Bars labeled with different letter scripts indicate statistically significant differences. CMC (*n* = 5), LET (*n* = 6), LET + HFD (*n* = 7), SO (*n* = 5), EV (*n* = 6), and EV + HFD (*n* = 5).

Fasting plasma insulin levels were evaluated in all groups to assess the presence of compensatory hyperinsulinemia. Interestingly, neither the LET nor the EV treatment induced any changes in insulin concentration (Figure 3a). The addition of an HFD treatment increased insulin levels; however, due to the high variability among experimental animals, it did not reach statistical significance (Figure 3a). Hyperandrogenism is the hallmark of PCOS, therefore plasma testosterone concentrations were measured in all experimental groups. As expected, the LET and LET + HFD groups exhibited a significant increase in testosterone (2.2- and 3.1-fold, respectively) compared to the CMC group (Figure 3b). On the other hand, while the EV group showed only a tendency for an increase in testosterone (1.8-fold increase for EV + HFD), it did not reach statistical significance (Figure 3b). LET + HFD had consistently higher testosterone levels compared to animals in the estradiol valerate model. Finally, we measured estradiol levels in plasma in the experimental groups. Unexpectedly, none of the treatments resulted in a significant decrease in estradiol concentration (Figure 3c).

**Figure 4:**
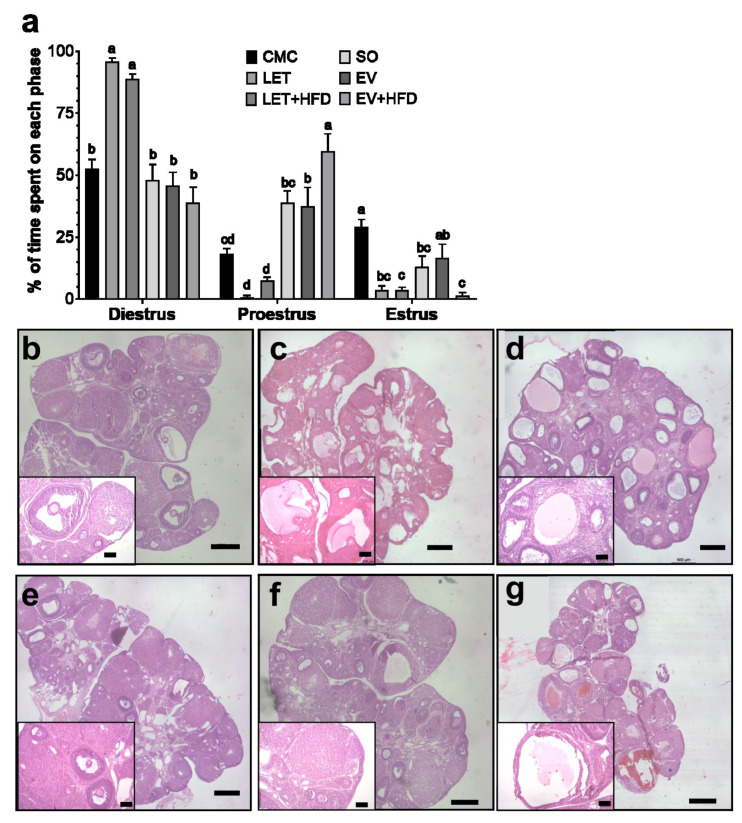
**Reproductive parameters of treated females.** (**a**) Percentage of time spent on each phase pf the estrus cycle determined by vaginal cytology. Representative images of ovarian sections of (**b**) CMC, (**c**) LET, (**d**) LET + HFD, (**e**) SO, (**f**) EV, and (**g**) EV + HFD groups. Each bar represents mean ± SEM. Bars labeled with different letter scripts indicate statistically significant differences. CMC (*n* = 6), LET (*n* = 7), LET + HFD (*n* = 12), SO (*n* = 6), EV (*n* = 8), and EV + HFD (*n* = 7). Bars in panels (**b**–**g**) represent 500 μm; inserts: 100 μm.

In addition to metabolic and endocrine alterations, PCOS is characterized by a series of reproductive abnormalities. To assess alterations in the reproductive cycle, vaginal smears were performed on all experimental groups during 21 consecutive days. Both the LET and LET + HFD groups displayed a significant increase in the time spent in the diestrus phase, while the proestrus and estrus phases were significantly reduced (Figure 4a). On the other hand, only the EV + HFD showed a significant decrease in the estrus phase, with a concomitant increase in proestrus (Figure 4a). Finally, the ovarian morphology was assessed in experimental animals by hematoxylin and eosin staining of paraffin sections. Both vehicle groups, CMC and SO, displayed normal ovarian morphology, including several growing follicles in several stages of development. Additionally, they exhibited multiple corpora lutea, indicating previous ovulations (Figure 4b,e). On the contrary, letrozole treatment induced multiple cystic structures (Figure 4c) that were more prominent with the addition of an HFD (Figure 4d). Furthermore, LET and LET + HFD showed few corpora lutea, indicating ovulatory dysfunction. On the other hand, estradiol valerate by itself did not modify ovarian morphology, growing follicles and corpora lutea were still observed, along with few cysts (Figure 4f). However, EV + HFD displayed multiple cysts; nonetheless, there were some corpora lutea still present (Figure 4g).

## 4. Discussion

PCOS is among the most common endocrine disorders affecting women of reproductive age. It is a highly heterogeneous condition with diverse phenotypes and clinical manifestations. Although it is primarily known as a reproductive disorder, PCOS is closely associated with significant metabolic disorders, many of which are exacerbated by obesity. Thus, PCOS should also be considered a metabolic disorder, with insulin resistance as a central feature [20]. Insulin resistance and its compensatory hyperinsulinemia are fundamental in driving the hyperandrogenism and reproductive alterations characteristics of PCOS. As such, an ideal animal model for studying PCOS should develop such characteristics. However, most animal models of PCOS (i.e., androgen administration in the form of testosterone propionate of dihydrotestosterone), only partially reproduce the complete symptomatology of the disorder. For instance, EV administered at a dose of 4 mg induced irregular cycles and polycystic ovarian morphology (PCOM) it did not alter sex hormones nor gonadotropin concentrations. Even in the case of leptin deficient mice, while they develop the metabolic and endocrine features of PCOS, they do not develop PCOM [9,21]. Although we have gained significant insight into this disorder, the need for a more appropriate model still persists. Most PCOS models could be categorized either into prenatal and postnatal models, each one with different characteristics and uses. In the prenatal models, pregnant dams are injected intramuscularly or subcutaneously with androgens in the last portion of pregnancy [9]. These models have been extensively used to assess the developmental–intergenerational origin of PCOS. However, as previously mentioned, they do not resemble the full spectrum of reproductive and metabolic disorders [21]. Conversely, in the postnatal models, females are treated with a variety of pharmacological and environmental factors to induce hyperandrogenism and most of the reproductive abnormalities [21]. In this paper, we compared two of the most widely employed postnatal PCOS models, letrozole and estradiol valerate, either by themselves and in the context of a high-fat diet.

In this paper, we observed a significant weigh increase concomitant with letrozole treatment, which was enhanced by the administration of an HFD. The 1 mg/kg per day dose was chosen as it has been extensively used to induce PCOS-like symptoms [21,22]. This finding aligns with multiple papers indicating that letrozole treatment increases body mass in different regimes. For instance, the implantation of a 90-day continuous release letrozole pellet (36 mg, daily dose of 400 μg) increased weight in rats as soon as the first week of treatment [18]. Additionally, other studies showed that rat oral letrozole administration (0.5 mg/kg) also induces increased body mass [11]. Moreover, it has been shown that rat letrozole treatment can induce most of the PCOS characteristics (including weight gain) as soon as after 21 days of a daily administration of 1 mg/kg oral letrozole [9]. However, in a mouse model, letrozole treatment (even in the presence of an HFD) failed to induce increased body mass [23]. On the other hand, the EV and EV + HFD groups showed a tendency for a decreased body mass. This is in contrast with some studies that have employed estradiol valerate as a PCOS model. Estradiol valerate has been shown to increase body mass when administered at 1 day-old [24], 45 days-old [25], and adult rats [26]. However, additional studies have shown that estradiol valerate induces a significant weight reduction in Wistar rats [27]. The proposed mechanism for this phenomenon is an activation of the sympathetic nervous system, which in turn increases basal metabolic rate and fat consumption. Another study observed growth arrest in rats administered 2 mg of EV intramuscularly. These rats had a reduced food intake on days 4–12 post-injection; nevertheless, no differences in food consumption were observed in the subsequent 49 days. Moreover, by day 61 post-injection, EV-treated rats were hyperleptinemic [28]. Moreover, additional articles have shown that EV is capable of inducing growth arrest in a dose-dependent manner in female rats [29,30,31]. Thus, while LET has been consistently shown to increase body mass, EV has been shown to have a more varied response. However, one important caveat is that weight gain per se is not directly responsible for the enhanced metabolic dysregulation observed in PCOS, but rather an increase in adiposity [32]. Thus, adiposity was assessed by determining the relative weight of adipose tissue depots. The LET + HFD group displayed a consistent increase in the proportion of gonadal and retroperitoneal visceral adipose tissue, compared to the CMC group and the EV models. Interestingly, most studies report obesity as increased body mass, without differentiating between lean mass and adipose tissue. Mannerås and cols. (2007) reported that letrozole induced an increase in adipose tissue depots; however, when adjusted to the total weight of the animal, only parametrial fat was proportionally heavier in letrozole treated rats [22]. On the other hand, none of the groups in the EV model had any significant increase in adipose tissue mass, not even in combination with an HFD. This agrees with a previous report that EV reduced the weight of parametrial adipose tissue, probably due to enhanced lipolysis [28]. In fact, there is evidence in support for this hypothesis. A single dose of estradiol benzoate not only reduces the size of parametrial fat, but it also increases the activity of lipoprotein lipase in rats [33]. Therefore, letrozole performs significantly better at inducing increased body mass and adiposity, in a more consistent manner.

As previously noted, the metabolic aspects of PCOS are a significant part of its pathophysiology. First, fasting plasma glucose was assessed in experimental animals. While letrozole treatment showed a tendency for increased plasma glucose, it did not achieve statistical significance. This is consistent with previous studies in which a 1 mg/kg letrozole treatment, similar to the one employed in this study, did not induce a significant change in fasting plasma glucose [9,34]. The addition of an HFD showed a potential enhancing effect over LET; although, it did not reach statistical significance. Previous research shows that an HFD induces increased plasma glucose in rats treated for 12 weeks with letrozole, a significantly longer treatment compared to our 21-day regime [11]. Therefore, fasting hyperglycemia could be achieved by an extended letrozole regime combined with HFD. The same pattern was observed in the EV and EV + HFD groups. The literature reflects conflicting findings regarding fasting glucose concentrations in studies employing EV. For example, one study has shown increased fasting plasma glucose, 30 days after a 5 mg/kg EV administration, higher than the 4 mg/kg employed in this study [35]. While other studies showed increased plasma concentrations 60 days after PCOS induction, employing a single 2–4 mg/kg dose [36,37]. Nevertheless, it is worth mentioning that both studies employed adult rats (200 g of body weight), compared to our model which employed 21-day-old rats. On the other hand, other studies failed to find any changes in glucose homeostasis in adult rats treated with EV, even in the presence of an HFD [38,39]. Regarding glucose tolerance, the LET + HFD group displayed a consistent increase in the first 90 min, compared to LET alone. Furthermore, the area under the curve (AUC) of the LET + HFD group was significantly increased; contrary to the LET and EV groups. It has been consistently observed that LET + HFD impairs glucose tolerance, compared to LET and control groups [11,34,40]. On the case of insulin sensitivity, the LET and LET + HFD groups did not show any significant changes, compared to the CMC group. There are some discrepancies regarding the induction of insulin resistance with letrozole treatment. For example, while some studies have not shown differences in insulin sensitivity after a 90-day letrozole treatment through a continuous-release pellet providing a daily dose of 400 μg [22], others have shown a significant reduction in insulin sensitivity after 6–12 weeks of LET + HFD treatment [11,34]. Thus, it is likely that the lack of any changes in insulin sensitivity are due to a relatively short (21 days) letrozole treatment. In the case of the EV model, while there was a tendency for an increased insulin sensitivity, it was not statistically significant. This result is in line with previous studies that have shown a lack of insulin resistance in animals treated with estradiol valerate [9,22]. Furthermore, there is evidence that hyperandrogenism in combination with an HFD disrupts insulin sensitivity [41]. Since EV treatment did not induce hyperandrogenism, this could be an explanation for the normal insulin sensitivity observed.

Dyslipidemia is a common feature of women with PCOS [42]; hence, the plasmatic concentrations of triacylglycerols and cholesterol were analyzed in experimental animals. Surprisingly, none of the treatments increased lipid plasma concentrations, even in the presence of an HFD composed primarily of saturated fats from animal origin. As previously mentioned, there are some discrepancies in the phenotypes observed in the most common PCOS models. For example, some studies have found that a 10-week letrozole treatment does not induce dyslipidemia [43], while a 12-week letrozole–HFD treatment only increases triacylglycerols [11]. Others have shown only an increase in total cholesterol and LDL–cholesterol (LDL–C) after LET + HFD treatment [35]. A similar study to this found that an 8-week letrozole treatment in combination with an HFD only induced an increase on triacyclglycerols without altering total cholesterol levels; the proposed mechanism was a dysbiosis in the gut microbiome [44]. Furthermore, a metanalysis on letrozole over the lipid profile on postmenopausal women showed that letrozole treatment induced a modest reduction on HDL–C and total cholesterol, while it did not affect triacyclglycerols and LDL–C [45]. On the other hand, estradiol valerate has been shown to increase triacylglycerols, total cholesterol and LDL–C [25]. Interestingly, a study showed that EV administration in aged rats (20 months) improved lipid profile [46]. Thus, the LET and EV regimes tested in this study might not be suitable for the characterizations of dyslipidemia in PCOS. Lastly, in order to further characterize the metabolic disturbances, fasting plasma insulin was determined in all treatments. PCOS patients often develop a compensatory hyperinsulinemia as a consequence of insulin resistance; furthermore, it is widely considered that hyperinsulinemia is a major driver of hyperandrogenism in PCOS [47]. Interestingly, neither the LET nor the EV group had increased insulin levels; however, the inclusion of a HDF showed a tendency for increased insulin concentrations in both groups. This is consistent with previous reports that show that an HFD can enhance the metabolic dysregulation caused by the pharmacological treatment [25,34,37]. It is worth noting that although LET + HFD had a 4.3-fold increase compared to the CMC group, due to the high variability among the experimental animals, it did not reach statistical significance. Therefore, the administration of an HFD in addition to the pharmacological model of PCOS enhances the metabolic disturbances.

Aromatase is the rate-limiting reaction in the conversion of androgens to estrogens; thus, aromatase inhibitors have been extensively used to suppress endogenous estradiol production. Letrozole is a potent third generation aromatase inhibitor that has been consistently used to block endogenous estradiol production in rats [9,34,48]. As expected, letrozole treatment significantly increased plasmatic testosterone concentrations. Interestingly, the addition of HFD tended to amplify letrozole effect, which has also been reported by other investigators [11]. Furthermore, the testosterone-increasing effect of letrozole was significantly superior to that of estradiol valerate. In contrast, previous studies have shown less consistent results with estradiol valerate. Some have shown a two-fold significant increase in plasma testosterone [36,37], whereas others have shown only a ~30% increase [49], or no changes at all [35,38]. Therefore, for the investigation of hyperandrogenism related phenomena, letrozole treatment has been shown to give better and more consistent results. Unexpectedly, there were no changes in circulating estradiol concentrations in the letrozole treated groups. This is in line with other reports that show a non-significant reduction in estradiol concentration [34]. There is a previous report that has shown that letrozole significantly reduce estradiol levels in the ovary without altering circulating levels; they proposed that a reduction in uterine weight could be a viable bioassay for estrogen effect [48]. Concordantly, we observed a significant reduction in uterine weight in LET treated rats (Supplementary Figure S1). In addition to hyperandrogenism, two of the defining characteristics of PCOS are oligoanovulation and polycystic ovarian morphology [2]. Both LET and LET + HFD displayed alterations in estrus cycle and spent a significantly longer time in the diestrus phase and less time in proestrus and estrus as previously reported [22]. On the other hand, only EV + HFD displayed a reduced duration of the estrus phase, similar to previous reports [38]. Finally, letrozole by itself or with the addition of an HFD displayed follicular cysts, similar to what has been previously reported [21]. On the contrary, while estradiol valerate induced cyst formation, it was inferior to that of letrozole. In summary, we tested two of the most widely employed pharmacological models of PCOS by themselves or in the presence of an HFD. While both induced the reproductive characteristics of PCOS, letrozole has been consistently shown to produce the metabolic and reproductive alterations often associated with PCOS. In both instances, the administration of an HFD enhanced some of these dysregulations; therefore, it could be a useful method for an improved PCOS model.

## 5. Conclusions

This study evaluated two commonly used postnatal animal models of polycystic ovary syndrome (PCOS), letrozole and estradiol valerate (EV), with and without the addition of a high-fat diet (HFD). While both models successfully induced the hallmark reproductive features of PCOS, such as disrupted estrous cycles and polycystic ovarian morphology, only the letrozole model consistently replicated the metabolic disturbances associated with the condition. Letrozole plus HFD treatment led to significant weight gain, increased gonadal and retroperitoneal adiposity, impaired glucose tolerance, and elevated testosterone levels. In contrast, the EV model showed more variable results, especially regarding metabolic outcomes. These findings support the use of letrozole, particularly in combination with a high-fat diet, as a more robust and comprehensive model for studying both the reproductive and metabolic aspects of PCOS. This model may be valuable for future investigations into the pathophysiology of PCOS and the development of therapeutic strategies.

## Data Availability

The data presented in this study are available upon reasonable request from the corresponding author.

## Supplementary Materials

The following supporting information can be downloaded at: https://www.mdpi.com/article/10.3390/biology14060592/s1, Figure S1: Uterine weight of treated females; Figure S2: Vaginal cytology; Table S1: *p* values for each individual time point in the weekly body weight gain. Any comparison not shown is not statistically significant; Table S2: *p* values for each individual time point in the intraperitoneal glucose tolerance test. Any comparison not shown is not statistically significant; Table S3: *p* values for each individual time point in the intraperitoneal insulin tolerance test. Any comparison not shown is not statistically significant.
