# Supplementary material for: Comparative Analysis of Letrozole and Estradiol Valerate PCOS Models: Reproductive and Metabolic Outcomes with and Without High-Fat Diet
Source: Biology (Basel). 2025 May 23;14(6):592. doi: 10.3390/biology14060592 (PMC12189083; doi:10.3390/biology14060592)
Supplement: Supplementary file 1 [file biology-14-00592-s001.zip › biology-3629371-supplementary.pdf]

Table S1. *p* values for each individual time point in the weekly body weight gain. Any comparison not shown is not statistically significant.

Week	Comparison	*p* Value	Means (g)
**15**	CMC vs LET + HFD	0.0001	240.4 vs 289.4
	LET vs EV + HFD	0.0102	266.6 vs 229.6
	LET + HFD vs EV	0.0001	289.4 vs 241.0
	LET + HFD vs EV + HFD	0.0001	289.4 vs 229.6
	SO vs EV + HFD	0.0246	266.4 vs 229.6
**14**	CMC vs LET + HFD	0.0041	249.3 vs 284.5
	LET vs LET + HFD	0.0078	249.9 vs 284.5
	LET + HFD vs EV	0.0001	284.5 vs 232.4
	LET + HFD vs EV + HFD	0.0001	284.5 vs 231.1
	SO vs EV + HFD	0.0353	265.7 vs 232.4
**13**	LET + HFD vs EV	0.0163	266.6 vs 234.4
	LET + HFD vs EV + HFD	0.0012	266.6 vs 224.3
**12**	CMC vs EV	0.0083	245.5 vs 206.8
	LET vs LET + HFD	0.0273	220.0 vs 252.4
	LET + HFD vs EV	0.0003	252.4 vs 206.8
	LET + HFD vs EV + HFD	0.0102	252.4 vs 216.3
	SO vs EV	0.0322	242.8 vs 206.8
**11**	LET vs EV	0.0049	246.3 vs 204.0
	LET + HFD vs EV	0.0053	241.0 vs 204.0
	SO vs EV	0.0464	238.5 vs 204.0

Table S2. *p* values for each individual time point in the intraperitoneal glucose tolerance test. Any comparison not shown is not statistically significant.

Time point	Comparison	*p* Value	Means (mg/dL)
**15 min**	LET vs LET + HFD	0.007	326 vs 452
	LET + HFD vs SO	0.0137	452 vs 358
	LET + HFD vs EV	0.0038	453 vs 324
	LET + HFD vs EV + HFD	0.0012	454 vs 311
**30 min**	CMC vs EV	0.0451	426 vs 317
	LET vs LET + HFD	0.0015	333 vs 473
	LET + HFD vs EV	0.0002	473 vs 317
**60 min**	LET vs LET + HFD	0.0039	227 vs 362
	LET vs EV + HFD	0.0048	227 vs 371
	LET + HFD vs EV	0.0001	362 vs 196

	EV vs EV + HFD	0.0002	197 vs 371
**90 min**	CMC vs LET + HFD	0.0406	164 vs 237
	LET + HFD vs EV	0.0001	237 vs 123
	EV vs EV + HFD	0.0098	123 vs 213

Table S3. *p* values for each individual time point in the intraperitoneal insulin tolerance test. Any comparison not shown is not statistically significant.

Time point	Comparison	*p* Value	Means (% of basal)
**15 min**	LET vs EV + HFD	0.009	117 vs 84
**60 min**	LET + HFD vs EV + HFD	0.034	93 vs 54
**90 min**	CMC vs SO	0.0363	86 vs 50
	LET + HFD vs SO	0.0005	99 vs 50
**120 min**	CMC vs SO	0.007	95 vs 54
	LET vs LET + HFD	0.0096	78 vs 106
	LET + HFD vs SO	0.0001	106 vs 54
	LET + HFD vs EV	0.0391	106 vs 83
	LET + HFD vs EV + HFD	0.0365	106 vs 77
	SO vs EV	0.031	54 vs 83

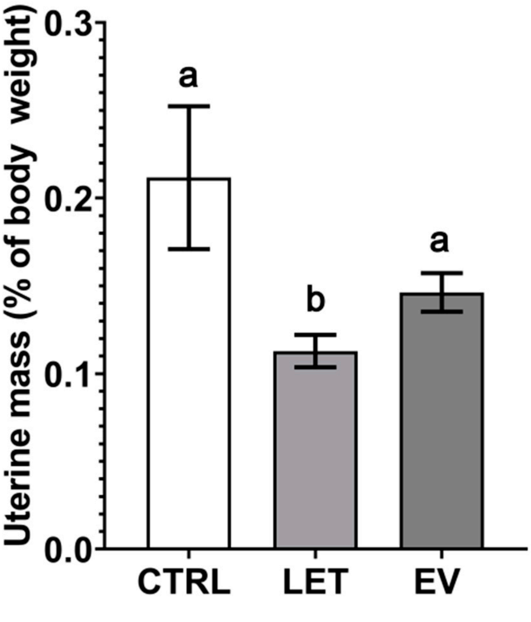

Supplementary Figure S1. Uterine weight of treated females. Weight of the uterus normalized to body weight. Each bar represents mean ± SEM. Bars labeled with different letter scripts indicate statistically significant differences. CTRL (*n* = 6), LET (*n* = 6) and EV (*n* = 7).

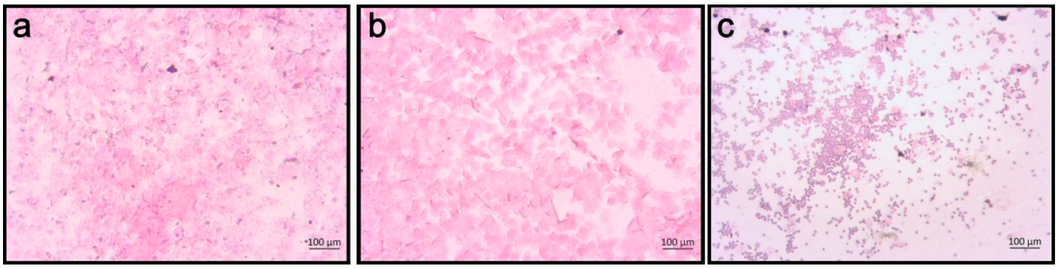

Supplementary Figure S2. Vaginal cytology. Representative images of vaginal cytology of rats in: a) Proestrus, b) Estrus and c) Diestrus. Bars represent 100 µm.
